# Facile and Ultrasensitive Determination of 4-Nitrophenol Based on Acetylene Black Paste and Graphene Hybrid Electrode

**DOI:** 10.3390/nano9030429

**Published:** 2019-03-13

**Authors:** Quanguo He, Yaling Tian, Yiyong Wu, Jun Liu, Guangli Li, Peihong Deng, Dongchu Chen

**Affiliations:** 1 School of Life Sciences and Chemistry, Hunan University of Technology, Zhuzhou 412007, China; hequanguo@126.com (Q.H.); tianyaling0212@163.com (Y.T.); wyy5082010@163.com (Y.W.); liu.jun.1015@163.com (J.L.); guangli010@hut.edu.cn (G.L.); 2 Key Laboratory of Functional Metal-Organic Compounds of Hunan Province; Key Laboratory of functional Organometallic Materials of Hunan Provincial Universities; Department of Chemistry and Material Science, Hengyang Normal University, Hengyang 421008, China; 3 School of Materials Science and Energy Engineering, Foshan University, Foshan 528000, China

**Keywords:** 4-Nitrophenol, nanomolar detection, water resources protection, graphene, acetylene black, voltammetric sensor

## Abstract

4-nitrophenol (4-NP) is a hazardous waste and a priority toxic pollutant identified by US Environmental Protection Agency (EPA). Hence, in this paper, a voltammetric sensor was proposed for the direct and sensitive detection of 4-nitrophenol (4-NP) at nanomolar level in complex matrices by using graphene and acetylene black paste hybridized electrode (GR/ABPE). Under optimal conditions, the calibration curve demonstrates a linear relationship for 4-NP in the range from 20 nM to 8.0 μM and 8.0 μM to 0.1 mM separately with the detection limit of 8.0 nM. In addition to it, the performance of the GR/ABPE in practical applications was evaluated by detecting 4-NP in various water samples, and satisfactory recoveries were realized. Therefore, GR/ABPE may have a great potential application for facile and sensitive detection of 4-NP in complex matrices at nanomolar level.

## 1. Introduction

Aromatic compounds containing nitro groups are important chemicals. They are widely used in the petroleum, wood, textile, pharmaceutical, paper-making, pesticides, dyes, and other industries. Generally speaking, the high toxicity of nitro-compounds, especially nitro-substituted aromatic compounds, is of particular concern, because these compounds have harmful effects on human beings, fish, aquatic organisms, and other life forms. Analyses of the nitro-aromatic compounds, especially nitrophenols, in waste waters have received considerable attention. Nitrophenol is a family of nitrated phenols with three isomers, 2-nitrophenol (2-NP), 3-nitrophenol (3-NP) and 4-nitrophenol (4-NP). However, the toxicity of nitrophenol isomers are different. 4-NP is highly toxic and exhibits a much more serious impact on the growth and metabolic activities of the organism [1], which can cause significant damages to biodegradation and the human health including methemoglobinemia: injuries to the liver and kidney. Excessive inhalation or acute intake of 4-NP by humans can cause a headache, lethargy, nausea, and even cyanosis. It can also damage the growth of microbes, animals, and plants [2]. Because 4-NP has obvious toxicity to the environment and human body, it has been listed as the priority pollutant of Environmental Protection Agency (EPA), which sets the permitted toplimit of 0.43 µM 4-NP in drinking water. There are scarcely-known natural sources of 4-NP in the environment. The anthropogenic sources of 4-NP in the atmosphere, soil, and water may attribute to industrial manufacturing and processing. For examples, waste water from various industries such as foundries, steel manufacturing, pharmaceutical processing and production and electrical/electronic components manufacturing may also release 4-NP into the surface water. In addition, 4-NP is difficult to degrade significantly by traditional treatments. Consequently, an efficient, rapid, and ultrasensitive method is needed for trace 4-NP detection from all sources, especially water samples in various complex matrices.

At present, many methods or sensors for the detection of 4-NP have been studied. For example, high performance liquid chromatography (HPLC) [3,4], spectrophotometry [5], fluorimetry [6], competitive flow immunoassay [7], enzyme-linked immunosorbent assays (ELISAs) [8], and different electrochemical methods [9,10,11,12,13,14,15,16,17,18,19]. Although the chromatographic methods can offer good selectivity and detection limit, they often require time-consuming detection process and complex pre-treatment steps. Moreover, these instrumentations are rather complicated, expensive, and are hardly employed for on-site measurement. Spectrophotometry and fluorimetry either suffer from low sensitivity, narrow linear range, or high detection limit. In recent years, ELISAs for the determination of 4-NP have also been reported. However, the use of immunosensors is less advantageous because the stability of the biological material is low, complicated multistage steps are often required, large and expensive equipment is needed, and specific antibodies from killed animals or particular proteins obtained by recombinant techniques are required. Among these various detection techniques, electrochemical methods are the most attractive ones and have been drawing more and attention for their merits of simple operation, rapid response, cost-saving, less time required, and real-time detection. How to further improve and optimize the sensitivity and selectivity, decrease the detection limit of these electrochemical methods, many chemically modified electrodes have been described in previous reports [9,10,11,12,13,14,15,16,17,18,19]. Table 1 summarized the comparison and advantage data among different modified electrodes towards 4-NP detection. Each approach has its particular sensitivity and is subject to various limitations. So, there is still a need for the development of reliable electrochemically based sensors for the determination of 4-NP which are superior in accuracy, precision, and speed at the levels commonly encountered in different natural samples. 

Graphene (GR) is thin plane sheet dimensionally with sp^2^-hybridized carbon atoms, forming a flat hexagonal lattice. Since its discovery by Geim et al. in 2004 [20], GR has attracted wide attention for its excellent electrical conductivity, large surface area, excellent mechanical strength, and low production cost. Endowed with these outstanding physical and chemical properties and advantages, GR has become an attracting alternative component to develop and build up electrochemical sensors [21,22,23,24,25,26,27,28,29,30,31]. In recent years, great sign of progress has been made in GR based nanocomposites for 4-NP detection due to their synergy among the components used. For example, Kumar et al. fabricated a glassy carbon electrode (GCE) modified with polymerized 3,5-diamino-1,2,4-triazole@electrochemically reduced graphene oxide composite film and used it for sensitive detection of 4-NP [9]. Zhang et al. prepared a polycarbazole/N-doped graphene composite as electrode material which exhibited excellent electrocatalytic and adsorptive activities towards the reduction of 4-NP [10]. Ikhsan et al. synthesized reduced graphene oxide-silver nanocomposites via a facile one-step chemical reaction strategy, which showed good sensitivity and selectivity for 4-NP detection even at the level of nanomolar [11]. Jiao et al. prepared graphene–gold nanocomposites using the electrochemical co-reduction method. The oxidation response of 4-NP was significantly enhanced at the decorated GCE in comparison with the bare one and the gold nanoparticles modified one [12]. However, as far as we know, the preparation of electrochemical sensors which combine the excellent properties of acetylene black (AB) and GR for 4-NP determination has not been reported in any kinds of literature.

AB is a special carbon black usually produced via controlled combustion of acetylene under pressure in the air. AB has been widely used in electrochemical applications because of its large specific surface area, excellent conductivity and strong adsorption capacity. In previous work, our group has studied the electrochemical behavior of some electroactive substances using different acetylene black paste electrodes (ABPEs) [32,33,34,35,36,37,38,39]. ABPEs have been proved to have better performance according to reaction rate and reversibility than other conventional carbon electrodes, such as graphite electrode, carbon paste electrode, GCE, and carbon nanotube paste electrode. Furthermore, the background current is greatly restrained at ABPEs, which is favorable for its further applications in ultra-trace analysis.

In the present work, GR was immobilized on ABPE via drop-coating method. The electrochemical behavior investigation of 4-NP on the hybrid electrode was conducted in detail. Based on the unique and excellent properties of GR and AB, the fabricated modified electrode effectively facilitated the electron transfer of 4-NP, resulting in the increase of reduction signal as well as the determining sensitivity. Moreover, the GR/ABPE has some dominant advantages including easy fabrication, high stability, good reproducibility and low background current. Furthermore, the fabricated voltammetric sensor was used to detect 4-NP and give satisfactory results for various water samples in complex matrices. Based on these, a highly sensitive, facile and stable electrochemical sensor was established for the determination of 4-NP. 

## 2. Experimental

### 2.1. Chemicals and Solutions

Acetylene black (AB, 99.99% purity above) was provided by STREM Chemicals, USA. Graphite powder and 4-nitrophenol (4-NP) were purchased from Sinopharm Chemical Reagent Co. Ltd., Shanghai, China. Stock solution of 4-NP was prepared by dissolving in ethanol. Other chemicals with analytical reagent grade were purchased from Shanghai Chemical Reagent Co. Ltd., Shanghai, China, which can be used without further purification. 

### 2.2. Apparatus and Characterizations

Cyclic voltammetry (CV) and electrochemical impedance spectroscopy were recorded on an electrochemical workstation (CHI 660D, Chenhua Instrument Co. Ltd., Shanghai, China). Quantitative analysis was carried out on a polarographic analyzer (model JP-303E, Chengdu Instrument Factory, Chengdu, China) using second-order derivative linear sweep voltammetry. A traditional system consisting of three-electrodes was employed, including GR/ABPE with a diameter of 3 mm as the working electrode, platinum wire as the counter electrode, and saturated calomel electrode (SCE) as the reference electrode. All reported potentials were compared with SCE. The morphologies of different electrodes and materials were obtained on a JEOL JSM-6610LV scanning electron microscope (Jeol/Ntc., Tokyo, Japan). The pH measurements were conducted on a digital pH meter (pHs-3c, Shanghai Leichi Instrument Factory, Shanghai, China). High-performance liquid chromatography (HPLC) was performed on Waters model 510 system (Waters Ltd., Milford, MA, USA) comprising a Kromasil 100–5C18 (250 mm × 4.6 mm) column equipped with a Waters 2487 dual λ absorbance detector.

### 2.3. Synthesis of GR Dispersions

Graphene oxide (GO) was prepared from natural graphite powder through a modified Hummers method [40] and GR was obtained by reduction of GO with hydrazine [41]. Firstly, 23 mL concentrated sulfuric acid was cooled down to 0 °C, then 0.5 g graphite powder, 0.5 g sodium nitrate and 3 g potassium permanganate were consecutively added to the sulfuric acid. After heating for 2 h at 35 °C, the mixture reacted and turned greenish and pasty. When no more bubbling was observed, 40 mL water was slowly added into it. Afterwards, the paste was kept at 95 °C for around 30 min, then the mixture reaction was quenched carefully by an addition of 100 mL water. The mixture solution color turned from dark brown to bright yellow by a dropwise introduction of 3 mL hydrogen peroxide (30%). The resulting graphite oxide was filtrated as a yellow-brown filter cake, which was washed with both 1 M hydrochloric acid and water for several times, and then vacuum-dried at 50 °C for 24 h to obtain final graphite oxide powder. The powder (100 mg) was then re-dispersed in water (100 mL) and exfoliated under ultrasonication for 2 h, and any insoluble residue was centrifuged at 6000 rpm thus removed. The resulting stable GO solution (60 mL) was mixed with an addition of 100 µL hydrazine solution and following 950 µL 25 wt. % ammonia solution in a flask. After shaking violently, the flask was kept in a water bath (95 °C) with consistent stirring for 1 h. According to the above procedure, GR dispersions were prepared and they were used for further characterization and film fabrication.

### 2.4. Preparation of GR/ABPE

ABPE was prepared by the following steps. 3.0 g AB powder was mixed and ground carefully with 0.5 g solid paraffin in a mortar. The mixture was then heated to and kept at 80 °C to generate a homogeneous paste. A part of the paste is tightly packed into the cavity of a glass tube (inner diameter 3 mm), and an electrical contact was established between the copper wire and the end of the paste. The surface of ABPE was burnished on a weighing paper before use. Then 5.0 μL the GR-containing suspension was directly dropped onto the ABPE surface and dried at room temperature to obtain GR/ABPE. For comparison, the carbon paste electrode using graphite powder (CPE) and the GR modified CPE (GR/CPE) were also prepared by the similar procedures.

### 2.5. Electrochemical Measurement Procedure

All experiments were performed at room temperature, and typical voltammetric measurements are generally as follows. 0.1 M HCl and a standard 4-NP solution were both added into a 10-mL electrochemical cell to form the test solution, into which the three-electrode system was immersed then. Before determination, the solutions was deaerated by bubbling with nitrogen. CVs data were measured and collected between 0.6 V and −0.8 V at a scan rate of 0.1 V s^−1^. After 60 s accumulation at 0.0 V and a 5 s rest under stirring, second-order derivative linear sweep voltammetric data were also measured and collected over a potential span from 0.2 V to −0.8 V of a scan rate of 0.1 V s^−1^. The same procedure was also applied for various samples.

## 3. Results and Discussion

### 3.1. Electrochemical Characterization of the Modified Electrodes

**Figure 1:**
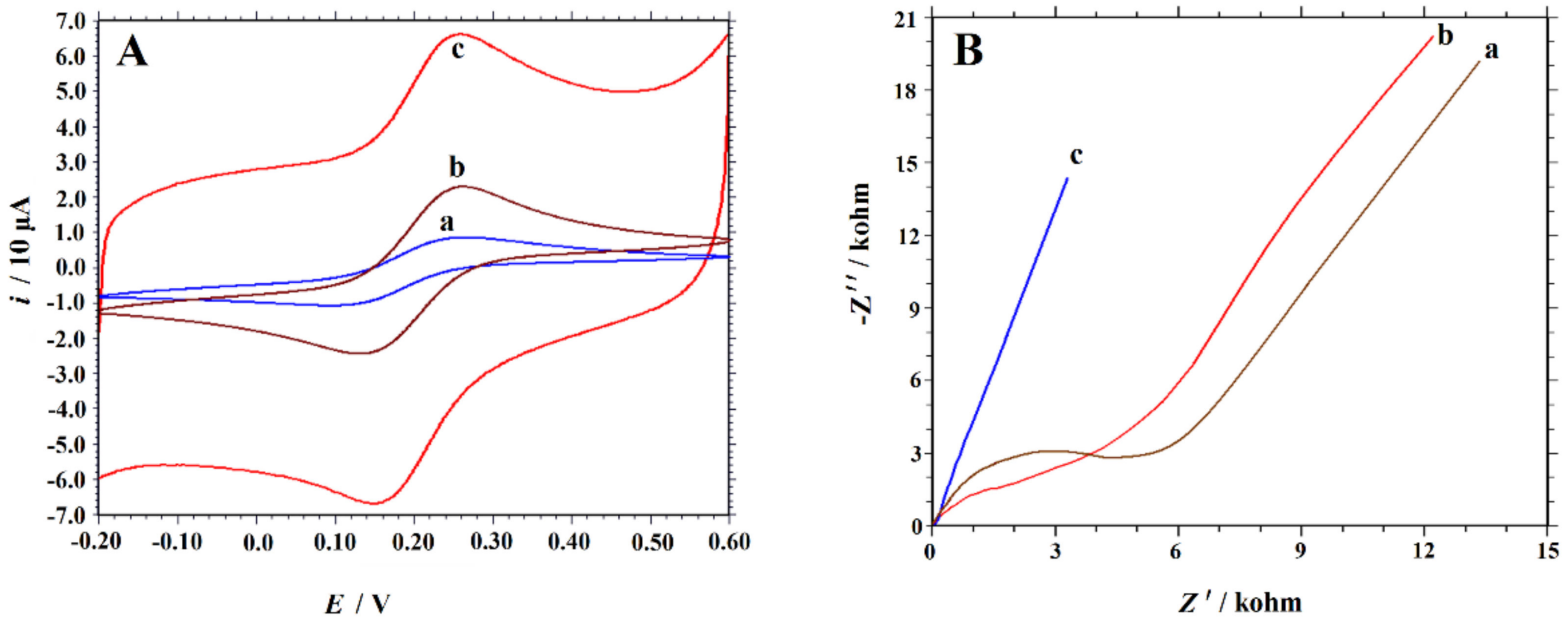
Cyclic voltammograms (**A**) and Nyquist plots (**B**) of different electrodes in 1.0 mM K_3_[Fe(CN)_6_] solution containing 0.5 M KCl. Scan rate: 0.1 V s^−1^. The frequency range was from 0.1 to 10^5^ Hz at the formal potential of 0.17 V. (a) CPE, (b) ABPE, (c) GR/ABPE.

The cyclic voltammetric responses of different composite electrodes were investigated in a mixed solution of 1.0 mM K_3_[Fe(CN)_6_] and 0.5 M KCl, and the corresponding results were plotted in Figure 1. On CPE (curve a), the potential peak-to-peak distance (Δ*E*_p_) recorded was 0.171 V and the redox peak current is small, implying a quasi-reversible electron transfer process involved in the electrochemical reaction. While on ABPE (curve b), there was observed a pair of well-defined redox peaks with significantly increased redox peak current and decreased Δ*E*_p_ to 0.132 V. This may be due to the large specific surface area, good conductivity, and strong electrocatalytic ability of AB, which leads to the increase of reaction sites on the surface of the electrode and promotes the electron transfer. On GR/ABPE (curve c), the redox peaks demonstrated the best reversibility (Δ*E*_p_ = 0.111 V) and the highest peak currents. The evident results indicated that GR can improve the electrode conductivity and is in favor of fast electron transfer. For further characterization of the modified electrode, electrochemical impedance spectroscopy was used. In electrochemical impedance measurement, the semi-circle diameter of impedance equals the electron transfer resistance (R_et_), which controls the electron transfer kinetics of the redox probe at the electrode surface. Figure 1B presented the Nyquist diagrams of CPE(a), ABPE(b), GR/ABPE(c) in 1.0 mM K_3_[Fe(CN)_6_] containing 0.5 M KCl. It can be seen that a big well defined semi-circle at higher frequencies was obtained at CPE, indicating large interface impedance. While the impedance value obtained at ABPE was smaller than that at CPE, which could be attributed to the conductivity and large surface area of AB, leading to a lower interface electron resistance. The further decrease of interface electron impedance was obtained at GR/ABPE, which demonstrated that GR was successfully immobilized on the ABPE surface.

### 3.2. Morphological Investigation

**Figure 2:**
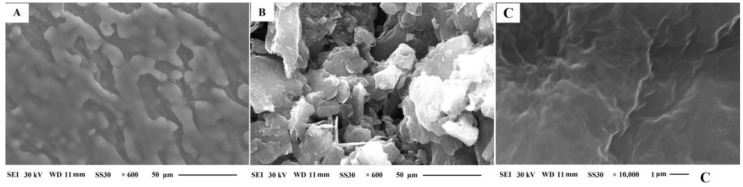
SEM images sampled from (**A**) acetylene black paste electrode (ABPE), (**B**) graphene and acetylene black paste hybridized electrode (GR/ABPE) at low magnification and (**C**) GR/ABPE at high magnification.

Scanning electron microscopy (SEM) was used to examine the morphologies of ABPE and GR/ABPE and the typical images were shown in Figure 2. From image of ABPE (Figure 2A), a granular surface was found with isolated and clearly distinguished granules. Figure 2B showed the SEM image of GR/ABPE. As can be seen the GR were immobilized onto the surface of ABPE and different morphology was observed for bare ABPE. The GR sheets tended to restack with one another, and the lateral size of the nanosheets ranged from several hundred nanometers to tens of micrometers in length. The typical wrinkled and crumpled GR sheets structure can be observed from the high magnification of the GR/ABPE (Figure 2C). The layered GR structure is helpful to maintain the high surface area of the electrode and to construct the electrochemical interface of 4-NP.

### 3.3. Electrochemical Behavior of 4-NP at GR/ABPE

**Scheme 1:**
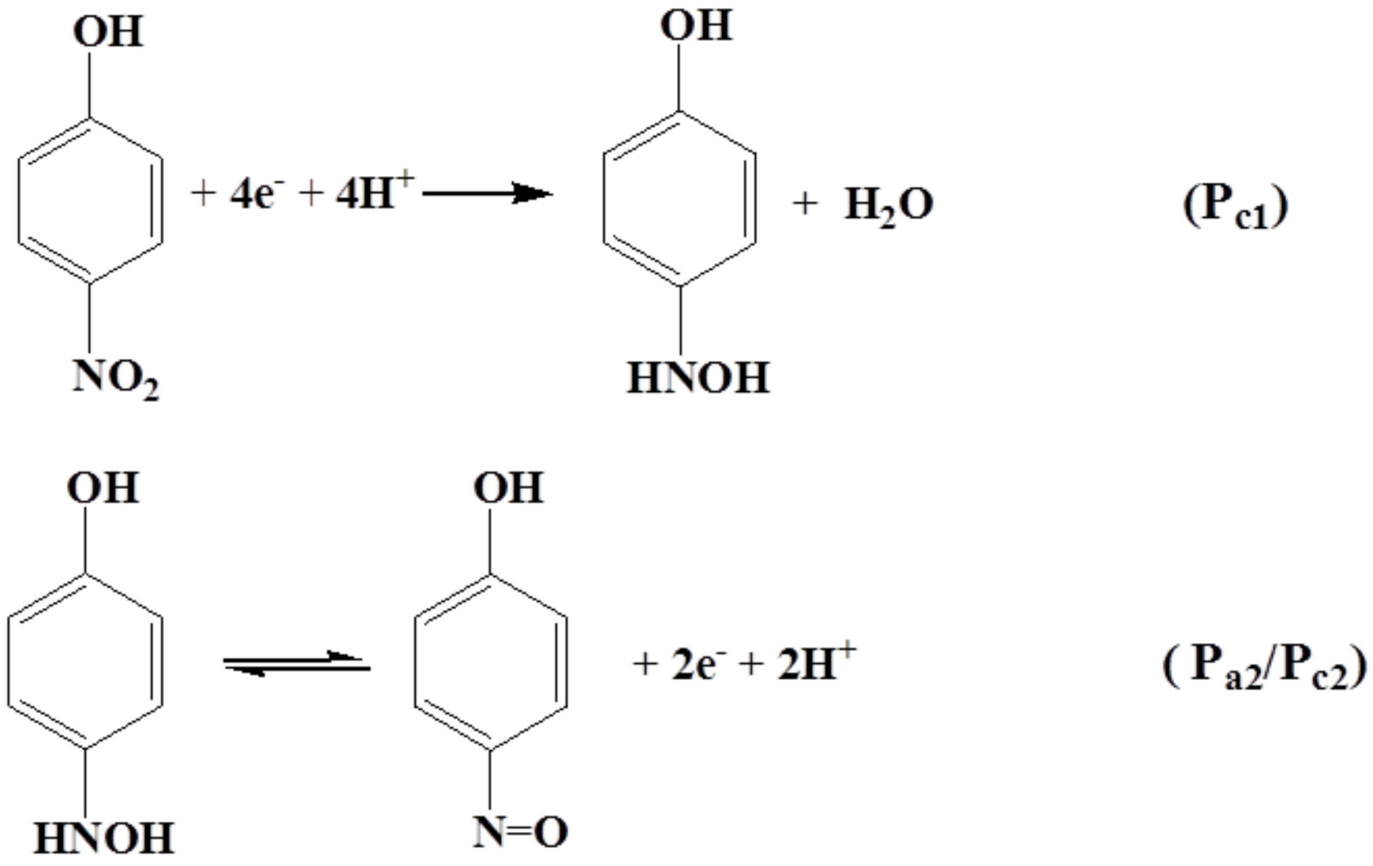
The eletrochemical reaction mechanism of 4-NP on the GR/ABPE.

**Figure 3:**
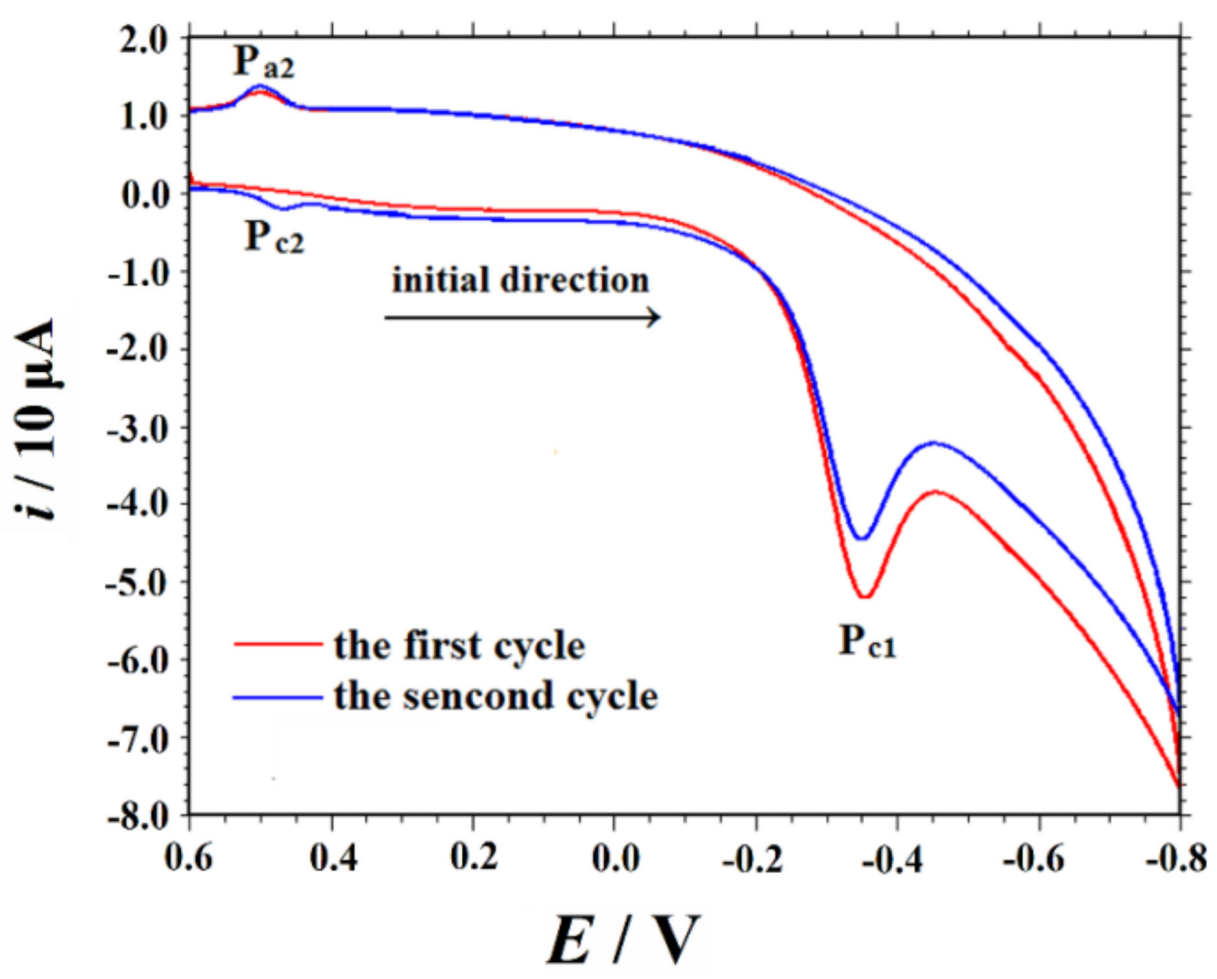
Cyclic voltammograms by continuous sweep cycles recorded on GR/ABPE (Concentration: 10 µM 4-NP in 0.1 M HCl solution, Scan rate: 0.1 V s^−1^).

In order to elucidate the electrochemical reaction mechanism of 4-NP on GR/ABPE, continuous cyclic voltammograms were adopted and recorded accordingly. As depicted in Figure 3, there was observed only a well-defined reduction peak (P_c1_) at −0.354 V in the first cathodic scanning step from 0.6 to −0.8 V. Under reverse scanning condition, there appeared an oxidation peak (P_a2_) at 0.499 V. During the second cathodic sweep, another reduction peak (P_c2_) at 0.409 V was observed, forming a redox couple with peak P_a2_. Meanwhile, the P_c1_ value of the second cyclic scan was significantly lower than that in the first cycle scan, which may be the attributed to the adsorption of 4-NP and its reduction products remaining on the surface of electrode, resulting in an inactivation of the electrode surface. In addition, if the initial cathodic scan is reversed from the start point before −0.354 V(that is, before the peak P_c1_), the redox couple P_c2_/P_a2_ will vanish. It implied evidently that the peak couple of P_c2_ and P_a2_ arose from the peak P_c1_ due to the reduction of 4-NP. As consistent as previous reports [12,42,43,44], the irreversible reduction (P_c1_) accorded with the direct reduction mechanism from nitrophenol to hydroxylaminophenol concerning a four-electrons and four-protons transfer process. While the reversible redox couple P_c2_/P_a2_ was characterized with a two-electrons and two-protons transfer process from hydroxylaminophenol electrogenerated to nitrosophenol. Therefore, the electron transfer mechanism can be described in the following equations illustrated in Scheme 1.

As obviously seen in Figure 3, the value of P_c1_ is higher than that of P_c2_ or P_a2_. In order to improve the sensitivity, P_c1_ was employed for the quantitative analysis of 4-NP in the following experimental studies.

**Figure 4:**
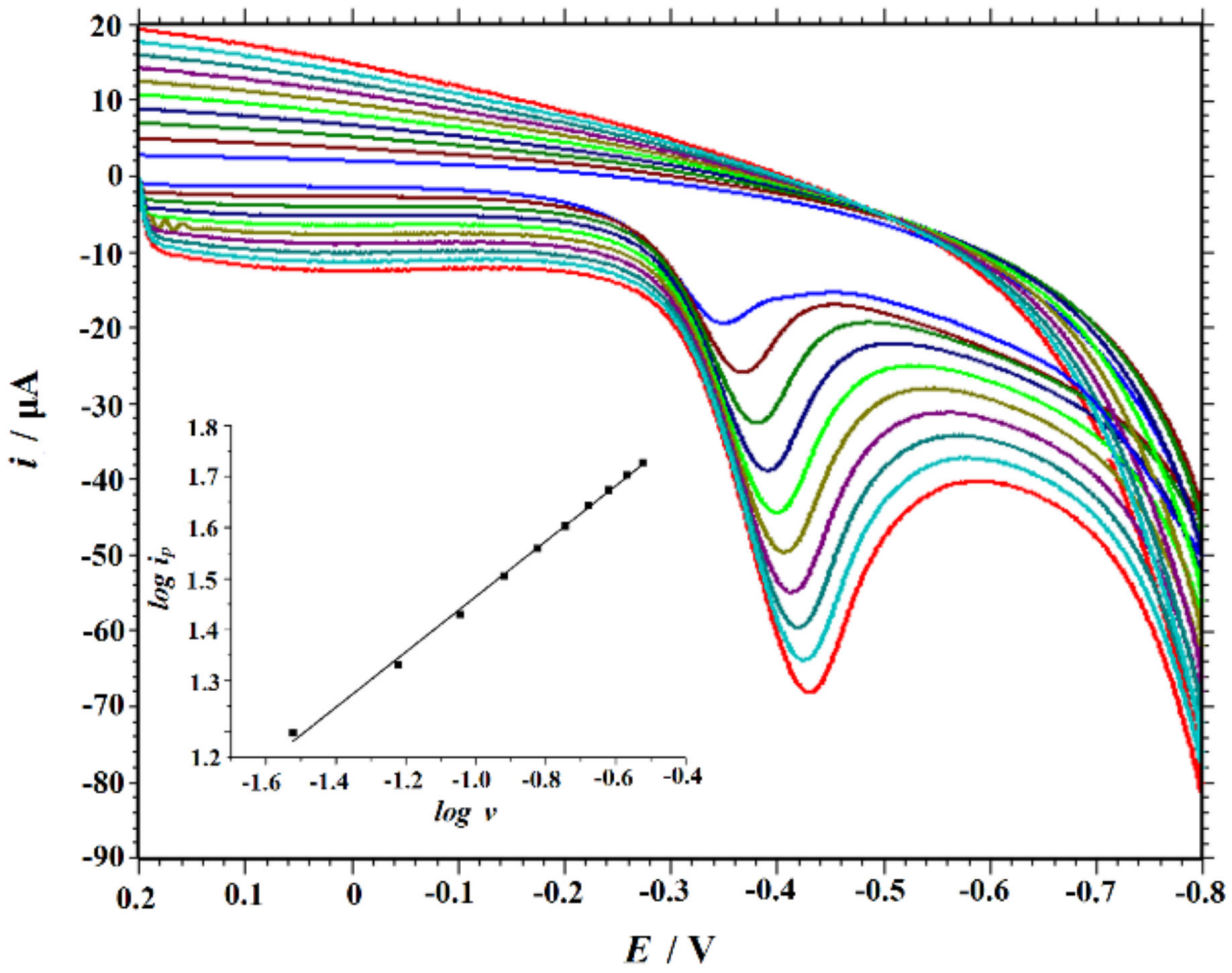
Cyclic voltammograms of GR/ABPE immersed in 10 µM 4-NP and 0.1 M HCl solution under different scan rates in the range 0.03 V s^−1^ to 0.3 V s^−1^; Inset: linear relationship fitting between log*i* and log*v*.

The effect of scan rate in the range from 30 to 300 mV/s on the electrochemical reduction of 10 µM 4-NP on GR/ABPE was illustrated in Figure 4. It was found that the reduction peak current gradually increased with the increasing of scan rate (Figure 4). A good linear relationship was shown between the peak current (*i*_p_) and the square root of scan rate (*v*^1/2^) with the regression equation as *i*_p_ (µA) = 10.256 *v*^1/2^ (V s^−1^) − 0.318(R^2^ = 0.9982), indicating explicitly the electrode process is diffusion control one. The diffusion controlled behavior was also cross-certified by plotting the data of log*i* versus log*v*, and a linear equation was obtained as follows: log*i* (µA) = 0.5417 log*v* (V S^−1^) + 1.0069 (R^2^ = 0.9978) (inset in Figure 4). Apparently, the slope of 0.5417 is close to 0.5, proving that the electrode process is of diffusion controlled nature [39].

### 3.4. Second-Order Derivative Linear Sweep Voltammetric Response of 4-NP

**Figure 5:**
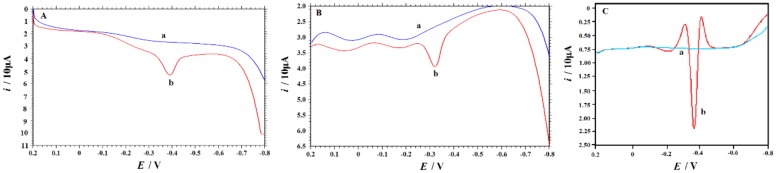
Linear sweep voltammetry (LSV) (**A**); differential pulse voltammetry (DPV) (**B**); and second-order derivative linear sweep voltammetry (**C**) of 0.1 M HCl (curve a) 0.1 M HCl containing 5.0 μM 4-NP (curve b) recorded on GR/ABPE after accumulated at 0.0 V for 60 s. Scan rate: 0.1 V s^−1^.

**Figure 6:**
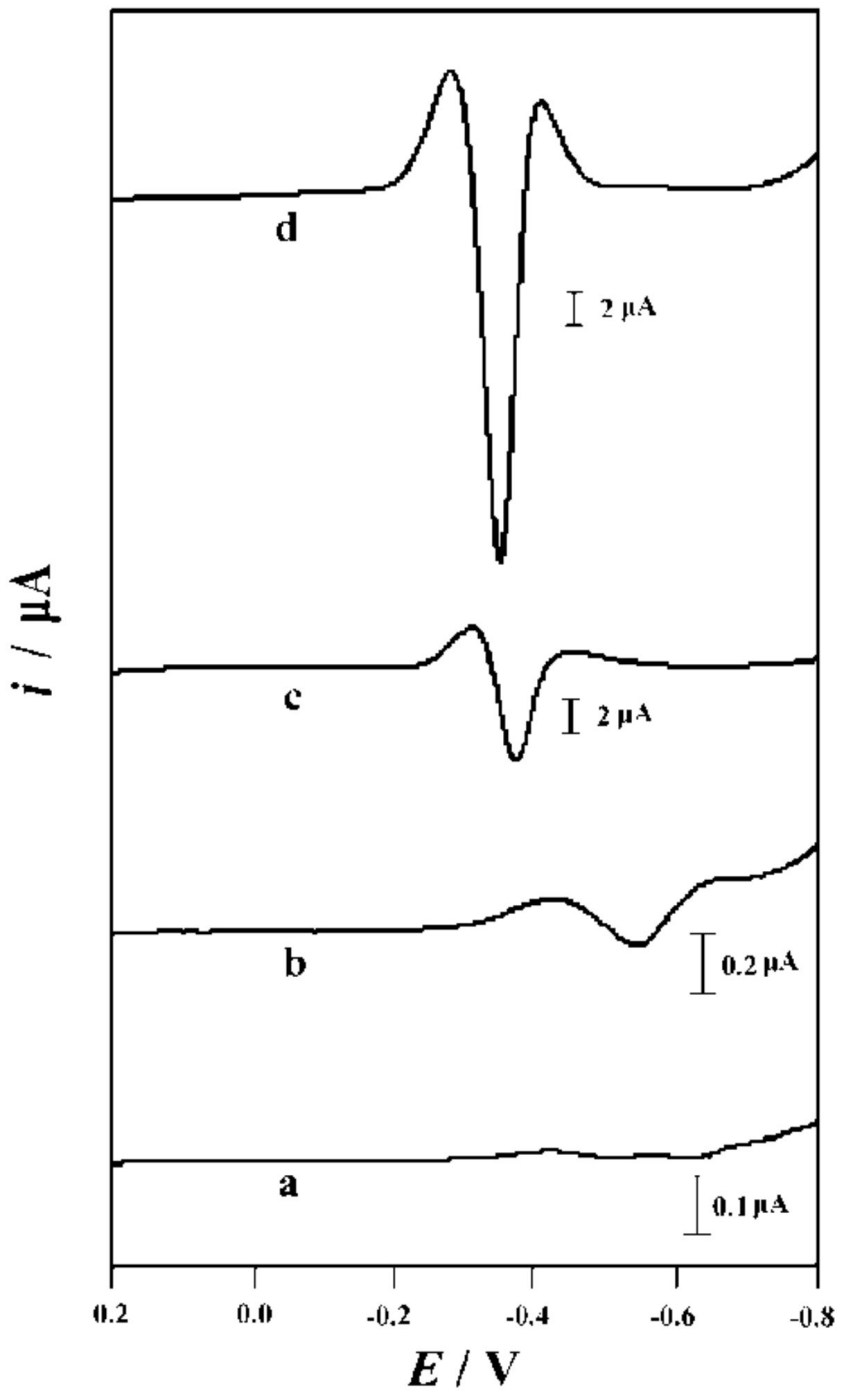
Second-order derivative linear sweep voltammograms recorded on (**a**) graphite powder (CPE), (**b**) ABPE, (**c**) GR/CPE and (**d**) GR/ABPE. (Concentration: 10 μM 4-NP and 0.1 M HCl, accumulation conditions: 0.0 V, 60 s, scan rate: 0.1 V s^−1^).

Second-order derivative linear sweep voltammetry is a widely used analytical technique for the enhancement of sensitivity and specificity in quantitative analysis [33,34,35,36,37,38,39]. This technology is the major advantage of JP-303 polarographic analyzer over other electrochemical analyzers. Compared with linear sweep voltammetry (LSV) and differential pulse voltammetry (DPV), as shown in Figure 5, second-order derivative linear sweep voltammetry has many advantages such as lower background current, larger peak current and higher resolution of overlapping. Thus, it was employed for the quantitative analysis of 4-NP. The electrochemical behavior of 10 μM 4-NP in 0.1 M HCl at different electrodes were investigated by second-order derivative linear sweep voltammetry after accumulating at 0.0 V for 60 s. The typical voltammograms of 4-NP at CPE (a), ABPE (b), GR/CPE (c), and GR/ABPE (d) were showed and compared in Figure 6, respectively. As expected, 4-NP exhibited a slow and very small cathodic peak (*i*_p_ = 0.01875 μA) on the bare CPE with the peak potential at -0.612 V, demonstrating a weak reduction of 4-NP. For ABPE, the peak current of 4-NP was larger than that of CPE (*i*_p_ = 0.2268 μA). Moreover, the peak potentials shifted positively to −0.536 V (curve b) with a characteristic of well-defined peak shape, which was attributed to the large specific surface area and strong electrocatalytic abilities of AB. When GR/CPE was used as working electrode, the reduction peak potential shifted positively (−0.384 V) accompanied with the oxidation peak current increased (*i*_p_ = 7.600 μA), suggesting the electron transfer reaction was promoted because of the unique properties of GR such as high conductivity and large surface area. While on the GR/ABPE electrode, the reduction peak current further increased (*i*_p_ = 29.42 μA) and the oxidation potential peak was transferred more positively (−0.352 V). The peak current on the GR/ABPE was about 4 times that on the GR/CPE and 130 times that on the ABPE. Undoubtedly, this performance should be contributed to the synergistic effect of GR and AB presented on the electrode surface. This remarkable current enhancement will allow the development of a highly sensitive electrochemical sensor for the determination of 4-NP.

### 3.5. Optimization of Electrochemical Determination Parameters

#### 3.5.1. Effect of the Amount of GR Modified onto Electrode Surface

The influence of the amount of GR modified onto electrode surface on the response of 4-NP was investigated. It was found that the 4-NP peak current increased gradually with increasing gradient amount of GR suspension from 0 to 5.0 μL. If the amount of GR suspension further increased from 5.0 μL to 15.0 μL, the reduction peak current of 4-NP decreased slightly. Moreover, the charging current increased with the enhancing amount of the GR suspension on the ABPE surface, preventing the determination of 4-NP at low concentration level. Accordingly, 5.0 μL of GR suspension was used for electrode modification.

#### 3.5.2. Effect of Supporting Electrolytes and Solution pH

**Figure 7:**
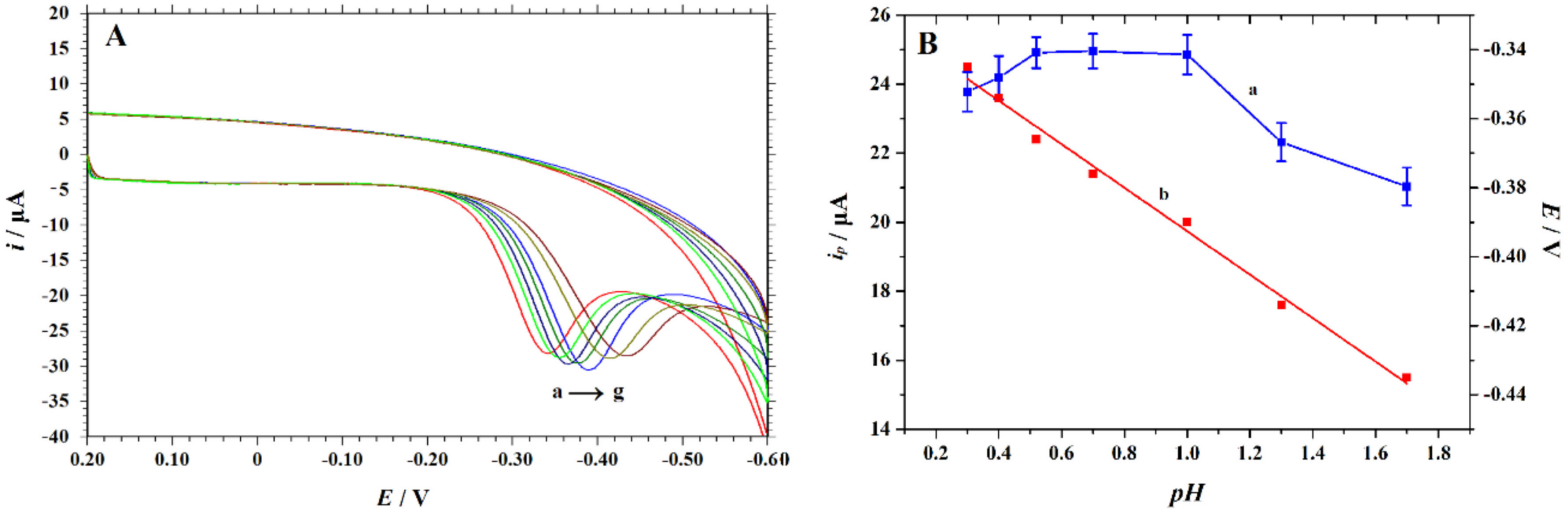
(**A**) Cyclic voltammograms recorded on GR/ABPE in 10 μM 4-NP and 0.1 M HCl solution with different pH values (from a to g are 0.30, 0.40, 0.52, 0.70, 1.00, 1.30, 1.70, respectively, scan rate: 0.1 V s^−1^); (**B**) the peak current and pH relationship while error bars represent SD, *n* = 3 (curve a); the linear relationship between the peak potential and pH (curve b).

Different supporting electrolytes were tested to determine how they affect the electrochemical responses of 4-NP, including Britton-Robinson buffer (pH 2.0~10.0), phosphate buffer (pH 3.0~9.0), (CH_2_)_6_N_4_-HCl buffer (pH 4.0~6.0), acetate buffer (pH 3.0~6.0), and many other acids and alkalis such as HCl, HNO_3_, H_2_SO_4_, HClO_4,_ and NaOH (each 0.1 M). It was observed that both the peak current and the voltammogram shape were best defined in HCl solution. Therefore, HCl was chosen as background solution for the electrochemical detection of 4-NP. The influence of solution pH on the voltammetric sensing performance of 4-NP was also examined. Hereby HCl was used as the supporting electrolyte, and varying pH values of solution were obtained by adjusting the HCl addition in the range of 0.02 M~0.5 M (pH 0.30~1.70) accordingly. All the cyclic voltammograms were depicted in Figure 7A. As shown in Figure 7B, the reduction peak current of 4-NP increased slowly with increasing pHs from 0.30 to 0.52, and the maximum peak current values were reached at pH 0.52~1.0. When the pH value is higher than 1.0, the decreased peak current was observed accordingly. With the increasing of solution pH, the peak potential changed negatively and a slope of −0.0631 V/pH was calculated based on the corresponding linear fitting, which was close to the theoretical value of −0.059 V/pH, indicating that the number of electrons and protons took part in 4-NP reduction was equal. This is in accordance with the electroreduction mechanism for 4-NP mentioned above.

#### 3.5.3. Accumulation Conditions

**Figure 8:**
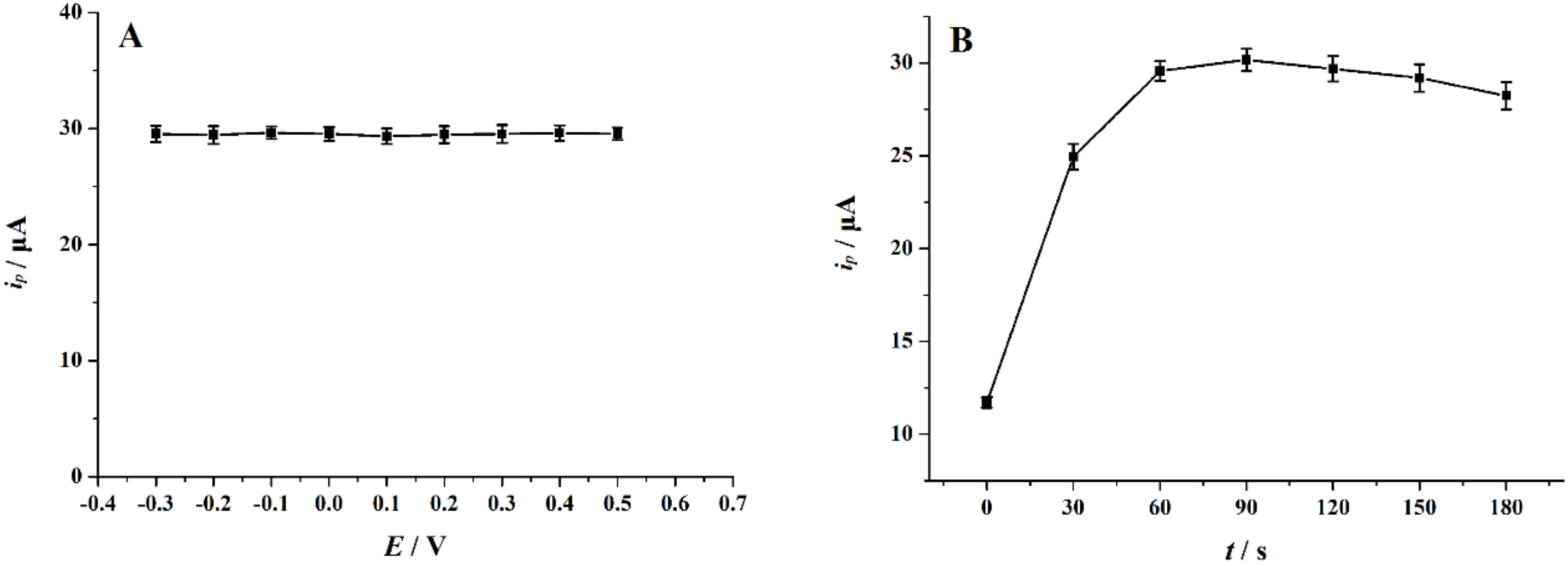
(**A**) Effect of accumulation potential and (**B**) accumulation time on the peak current of 10 µM 4-NP in 0.1 M HCl solution on GR/ABPE, error bars represent SD, *n* = 3; accumulation time was 60 s in Figure 8A.

To improve the sensitivity for 4-NP detection, accumulation is an effective and common way. The effect of accumulation potential and accumulation time on the electrochemical responses of 10 μM 4-NP on GR/ABPE were examined under stirring. The peak currents almost remain nearly unchanged at different accumulation potentials from −0.3 to 0.5 V, indicating that the accumulation potential has no direct influence on the electrochemical responses of 4-NP (Figure 8A). The peak current of 4-NP increased greatly as prolonging accumulation time within the first 60 s and then increased slowly (Figure 8B). Therefore, the optimal accumulation time and potential were selected as 60 s and 0.0 V, which were used throughout the experiments.

### 3.6. Calibration Curve

**Figure 9:**
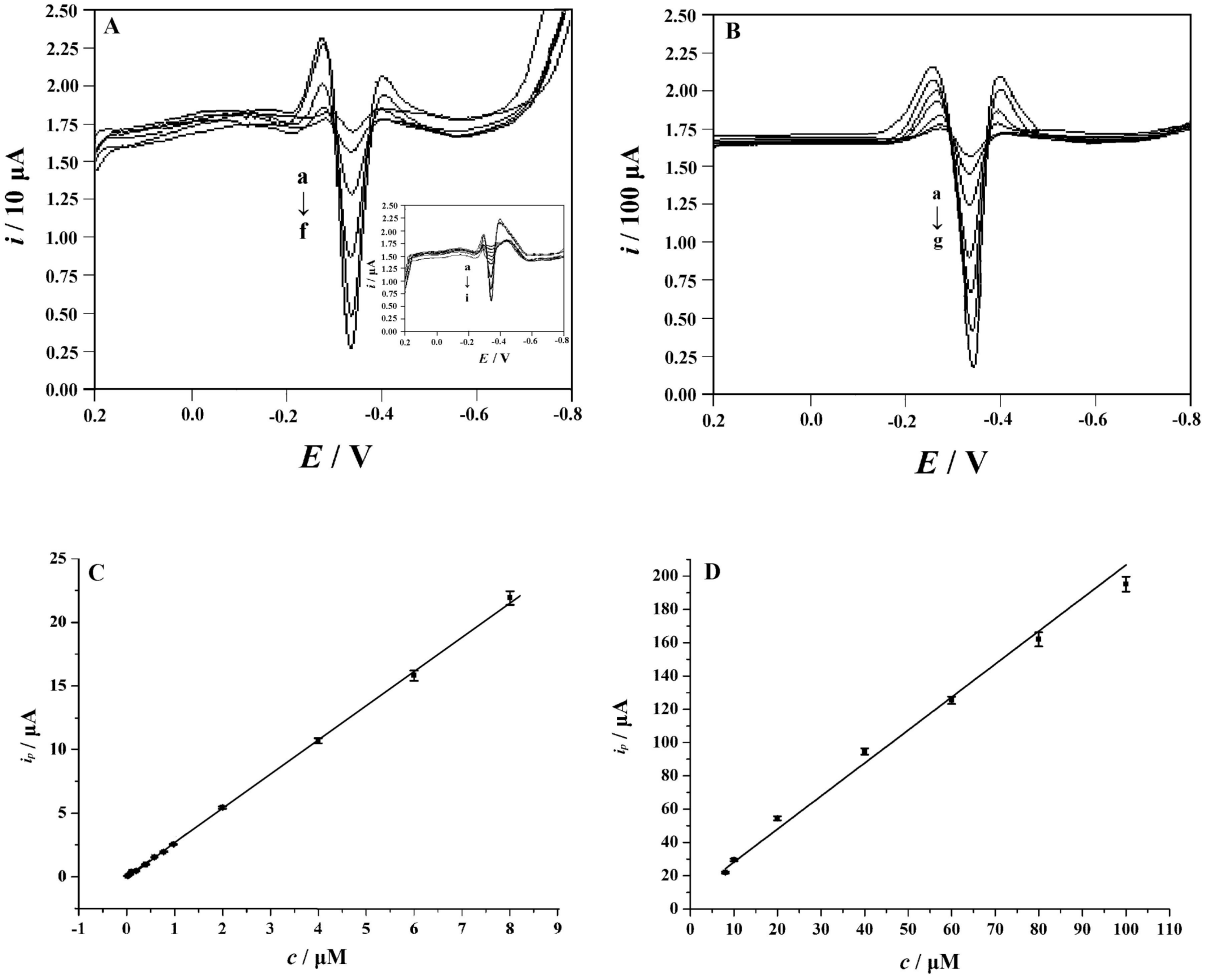
Second-order derivative linear sweep voltammograms collected on the GR/ABPE in various 4-NP concentrations and 0.1 M HCl solutions (**A**) Form a to f: 0.8, 1.0, 2.0, 4.0, 6.0, and 8.0 μM 4-NP (inset: form a to i: 0.02, 0.04, 0.06, 0.08, 0.1, 0.2, 0.4, 0.6, 0.8 μM 4-NP); (**B**) form a to g: 8, 10, 20, 40, 60, 80, and 100 μM 4-NP; (**C**) plots of the peak currents versus 4-NP concentrations in the range of 0.02~8.0 μM, and (**D**) plots of the peak currents versus 4-NP concentrations in the range of 8.0~100 μM, error bars represent SD, *n* = 3. Accumulation conditions: 0.0 V, 60 s, scan rate: 0.1 V s^−1^.

Under the optimized experimental condition, the calibration curve for 4-NP at GR/ABPE was characterized by second-order derivative linear sweep voltammetry. The typical voltammograms were described in Figure 9. The oxidation peak current is related to 4-NP concentration linearly in the range of 20 nM~8.0 μM and 8.0 μM~0.1 mM (inset of Figure 9). The linear regression equation can be described into two separate sections: ip (μA) = 2.717c (μM) −0.1574 (R^2^ = 0.9985) and ip (μA) = 1.8117c (μM) +14.424 (R^2^ = 0.9926), respectively. According to Özkan and Uslu [45], the detection limit (LOD) and the method quantitation limit (MQL) could be deduced from calibration line by using the equations LOD = 3 s m^−1^ and MQL = 10 s m^−1^, where s was the standard deviation of the measurements (six runs) and m was the slope of the calibration straight line in the low concentration range. Thus, the values of LOD and LQD calculated from the above equations were 8.0 nM and 26.7 nM, respectively. Table 2 listed the linear ranges and detection limits of different electrochemical sensors for 4-NP detection. Obviously, the GR/ABPE showed the widest linear range and much lower detection limit compared with other previous sensors [9,10,12,13,14,15,16,17,18,19]. The improved sensing performance can be attributed to the excellent combination and hybrid use of GR and AB described. Although rGO-Ag/GCE [11] provided lower detection limit, the dynamic linear range was rather narrow. Comparison of other significant aspects such as sensitivity, repeatability, electrode fabrication reproducibility, and storage stability have also been given in Table 1. It is found that the performances of GR/ABPE is comparable to or superior to the reported sensors. In addition, the preparation of other sensors were complicated with additional inclusion of functional materials. In our case, GR/ABPE exhibited many advantages such as simplifying electrode fabrication, lowing cost and saving time.

### 3.7. Repeatability, Reproducibility, and Stability of the Electrode

Seven successive assays at a single GR/ABPE were performed in a 1.0 μM 4-NP solution to evaluate the repeatability. The peak currents gave a relative standard deviation (RSD) of 2.75%. The reproducibility was estimated by preparing seven modified electrodes independently, which were applied for 4-NP detection with a RSD of 4.2%. The long-term stability of GR/ABPE was examined by detecting the current response of 1.0 μM 4-NP. The GR/ABPE was used daily and stored in the air. After a week, the current remained up to 94.7% of its initial value, demonstrating the good regeneration property and stability of GR/ABPE. After that, the current response began to decrease gradually. However, one month later the current signals still remained 87% initial response for 4-NP.

### 3.8. Interferences Test

In order to study the effect of possible substances that can be found in the water samples, various organic compounds and inorganic ions were studied. The peak current of 4-NP was detected three times. If the current change in the presence of other species was less than ± 5%, it would be considered that there is no interference on 4-NP detection. A great number of cations such as Cu^2+^, K^+^, Al^3+^, Zn^2+^, Ca^2+^, Mg^2+^, Pb^2+^, Cd^2+^, Ni^2+^, V^5+^, and Cr^3+^, and anions such as NO_3_^−^, SO_4_^2−^, and PO_4_^3−^ (each 1.0 mM) was added into 10 μM 4-NP, respectively. It turned out that 100-fold excess of these ions had no influence on the detection of 4-NP. Organic compounds were also examined. It was found that 100-fold concentrations of catechol, 2-aminophenol, phenol, 1,2-diaminobenzene, toluene, 2-chlorophenol, 2,4,6-trichlorophenol, 2,4-dichlorophenol, 50-fold concentrations of resorcinol, and 10-fold concentrations of 4-aminophenol, hydroquinone, did not interfere with the reduction signal of 10 μM 4-NP. However, 2-NP and 3-NP containing the same reductive groups can be reduced near the potential of 4-NP, which were found to affect the detection of 4-NP. However, their influence was not significant at low concentrations. Obviously, the interference of 2-NP and 3-NP can be neglected in the water samples which are not seriously polluted, such as rivers, lakes and tap water. If there are high concentrations of 2-NP and 3-NP in the water samples, such as industrial wastewater, the previous separation is necessary or chemometrics methods of analytical signals can be used to resolve the overlapped reductive peaks [46].

### 3.9. Quality Assurance and Quality Control for Water Samples

Whether the collected water samples are representative is an important part of water quality analysis, and whether the collection method is scientific and reasonable directly determines the final test results. In order to do a good job in quality control of water samples, the first is to strictly follow the principle of sampling and distribution, and the collected water samples truly reflect the quality of water sources in the region. Secondly, when preserving water samples, measures should be taken to avoid changes in water samples and to prevent external factors from affecting them. At the same time, they should be tested within the prescribed storage period. Water sample treatment is also very important. For example, the impurities in water samples are effectively removed by flocculation, precipitation and filtration, and the metal elements in water samples will not lost by acidification with nitric acid. The reagent blank test is also needed to prevent large deviation after treatment.

### 3.10. Practical Applications

To evaluate the precision and the accuracy of the developed method, GR/ABPE was applied to the detection of 4-NP in the real matrix samples. Before determination, various water samples (river water, lake water, tap water, and wastewater) were filtered to remove the solid materials. There was no 4-NP signal observed in the analyzed water samples, suggesting that no 4-NP exists in the water samples or the 4-NP concentration is under the limit of detection. Therefore, the method was applied to various samples distributed with different 4-NP concentrations. High-performance liquid chromatography (HPLC) was also used to detect the content of 4-NP to testify the accuracy of this method. The results were summarized in Table 3. It can be seen that the results obtained by the proposed method were in good agreement with the results obtained by HPLC. However, HPLC method needs more cost for reagent, on the contrary electrochemical method has many advantages such as low cost, easy fabrication and simple operation, so it is worthy of development. It is also clear that the recoveries of the developed method are in the range from 97.4% to 103.5%. These results implied that the sensor could be reliable and effective for 4-NP detection in environmental water samples.

## 4. Conclusions

The toxicity and environmental pollution by nitro aromatic compounds in water samples is the most recognized problem in worldwide. In this paper, a simple and effective method for trace analysis of 4-NP has been developed to protect water resources and food supplies. Herein, GR was used as a novel electrode modifier due to its many excellent properties, such as high specific surface area, upstanding electric conductivity and excellent electrochemical catalytic activity. ABPE can provide a favorable microenvironment for 4-NP and effectively accelerate the direct electron transfer rate of 4-NP at the electrode surface. Furthermore, the background current is greatly restrained at ABPE, which is favorable for its further applications in ultra-trace analysis. This study has led to the development of a high efficiency electrochemical sensor in environmental analysis with improved qualities such as: simplicity of electrode preparation, wide linear range, low detection limit, high selectivity, rapid regeneration, and long-term stability.

## Figures and Tables

**Table 1:** Performance comparison of different sensors for 4-nitrophenol (4-NP) detection.

Sensor	Sensitivity (μA/μM)	Repeatability (RSD%)	Fabrication Reproducibility (RSD%)	Storage Stability	Interferences	Recoveries for Samples (%)	Reference
^a^ pDAT@ERGO/GCE	0.0639; 0.0117	-	3.54	30 days	-	97.8–102.4	[9]
^b^ PCZ@N-GE/GCE	0.6549	-	3.5	5 days	2-nitrophenol, 3-nitrophenol	97.9%-104.5	[10]
^c^ rGO-Ag/GCE	19.83	-	-	7 days	-	99.7–102.3	[11]
^d^ GR-AuNP/GCE	0.1134	3.9	-	7 days	2-nitrophenol, 2,4,6-trinitrophenol	97.76–103.85	[12]
^e^ MWNT–Nafion/GCE	-	3.4	4.0	24 h	3-nitrophenol, 2,4-dinitrophenol, Cu^2+^, Pb^2+^, Fe^3+^	102.6–104.6	[13]
^f^ CHT-ZnO NDs/SPCE	0.1255	-	3.17	15 days	-	96.53–103.03	[14]
^g^ PMO/GCE	0.0819	-	3.1	10 days	Zn^2+^, Fe^2+^, Cu^2+^, phenol, 2-nitrophenol, 3-nitrophenol, 2,4-dinitrophenol, 4-chlorophenol, 2-chlorophenol, catechol, aniline	99.2–100.9	[15]
^h^ OMCs/GCE	0.156	-	-	3 weeks	-	97.2–98.8	[16]
^i^ CD–SBA/CPE	0.2465	0.91	-	-	cetyl trimethyl ammonium bromide, cetylpyridinium bromide, sodium dodecyl sulfonate, phenol, dihydroquinone	-	[17]
^j^ HA-NP/GCE	0.0295	-	3.54	30 days	phenol, 2-nitrophenol, hydroxyphenol	95.86–104.38	[18]
^k^ Gr-Chit/ABPE	1.4807; 0.9264	1.17	5.04	10 days	-	96.0–103.5 %	[19]
GR/ABPE	2.717, 1.8117	2.75	4.2	30 days	2-nitrophenol, 3-nitrophenol	97.4–103.5	This work

^a^ Electrochemical polymerized 3,5-diamino-1,2,4-triazole@electrochemically reduced graphene oxide composite film modificated on glassy carbon electrode; ^b^ polycarbazole/N-doped graphene modified glassy carbon electrode; ^c^ reduced graphene oxide-silver nanocomposite modified glassy carbon electrode; ^d^ graphene–gold nanocomposite film modified modified glassy carbon electrode; ^e^ gold nanoparticles modified pretreated graphite pencil electrode; ^f^ chitosan crafted zinc oxide nanoneedles modified screen printed carbon electrode; ^g^ poly(methyl orange) modified screen printed carbon electrode; ^h^ ordered mesoporous carbons modified glassy carbon electrode; ^i^ β-cyclodextrin-functionalized mesoporous silica (SBA-15) modified glassy carbon electrode; ^j^ hydroxyapatite nanopowder modified glassy carbon electrode; ^k^ graphene-chitosan composite modified acetylene black paste electrode.

**Table 2:** Comparison of Linear range and detection limit of the proposed sensor with other previously reported sensors.

Technique	Sensor	Linear Range (μM)	Correlation Coefficient (R^2^)	Detection Limit (μM)	Reference
^a^ DPV	pDAT@ERGO/GCE	5–200; 300–1500	0.97; 0.98	0.037	[9]
CV	PCZ@N-GE/GCE	0.8–20	0.997	0.062	[10]
^b^ SWV	rGO-Ag/GCE	0.01–0.1	0.998	0.0012	[11]
^c^ LSV	GR-AuNP/GCE	0.036–90	0.998	0.01	[12]
DPV	MWNT–Nafion/GCE	0.1–10	0.996	0.04	[13]
DPV	CHT-ZnO NDs/SPCE	0.5–400.6	0.995	0.23	[14]
DPV	PMO/GCE	0.6–10	0.991	0.17	[15]
DPV	OMCs/GCE	2–90	0.997	0.1	[16]
DPV	CD–SBA/CPE	0.2–1.4	0.997	0.01	[17]
DPV	HA-NP/GCE	1–300	0.9996	0.6	[18]
Second-order linear scan voltammetry	Gr-Chit/ABPE	0.1–20; 20–80	0.9996; 0.998	0.08	[19]
Second-order linear scan voltammetry	GR/ABPE	0.02–8.0; 8.0–100	0.9992,0.996	0.008	This work

^a^ Differential pulse voltammetry; ^b^ square wave voltammetry; ^c^ linear sweep voltammetry.

**Table 3:** Determination of 4-NP in water samples (*n* = 4).

Type of Water ^a^	Added (μM)	Found by This Method ^b^ (μM)	Recovery (%)	Found by HPLC ^b^ (μM)	Relative Error (%)
River	0.5	0.512 (±0.015)	102.4	0.483 (±0.012)	6.00
Lake	1.0	0.976 (±0.018)	97.6	1.024 (±0.022)	−4.69
Waste	2.0	2.071 (±0.033)	103.6	1.962 (±0.042)	5.56
Tap	0.1	0.102 (±0.002)	102.0	0.106 (±0.003)	−3.77

^a^ All water samples were collected from Hengyang, China. ^b^ average ± confidence interval, the confidence level is 95%.
